# Functional Dissection of Ipsilateral and Contralateral Neural Activity Propagation Using Voltage-Sensitive Dye Imaging in Mouse Prefrontal Cortex

**DOI:** 10.1523/ENEURO.0161-23.2023

**Published:** 2023-12-05

**Authors:** Pooja Gusain, Makiko Taketoshi, Yoko Tominaga, Takashi Tominaga

**Affiliations:** 1 Institute of Neuroscience, Tokushima Bunri University, Sanuki 769-2193, Japan; 2 Kagawa School of Pharmaceutical Sciences, Tokushima Bunri University, Sanuki 769-2193, Japan

**Keywords:** anterior cingulate cortex, corpus callosum, medial prefrontal cortex, voltage-sensitive dye

## Abstract

Prefrontal cortex (PFC) intrahemispheric activity and the interhemispheric connection have a significant impact on neuropsychiatric disorder pathology. This study aimed to generate a functional map of FC intrahemispheric and interhemispheric connections. Functional dissection of mouse PFCs was performed using the voltage-sensitive dye (VSD) imaging method with high speed (1 ms/frame), high resolution (256 × 256 pixels), and a large field of view (∼10 mm). Acute serial 350 μm slices were prepared from the bregma covering the PFC and numbered 1–5 based on their distance from the bregma (i.e., 1.70, 1.34, 0.98, 0.62, and 0.26 mm) with reference to the Mouse Brain Atlas (Paxinos and Franklin, 2008). The neural response to electrical stimulation was measured at nine sites and then averaged, and a functional map of the propagation patterns was created. Intracortical propagation was observed in slices 3–5, encompassing the anterior cingulate cortex (ACC) and corpus callosum (CC). The activity reached area 33 of the ACC. Direct white matter stimulation activated area 33 in both hemispheres. Similar findings were obtained via DiI staining of the CC. Imaging analysis revealed directional biases in neural signals traveling within the ACC, whereby the signal transmission speed and probability varied based on the signal direction. Specifically, the spread of neural signals from cg2 to cg1 was stronger than that from cingulate cortex area 1(cg1) to cingulate cortex area 2(cg2), which has implications for interhemispheric functional connections. These findings highlight the importance of understanding the PFC functional anatomy in evaluating neuromodulators like serotonin and dopamine, as well as other factors related to neuropsychiatric diseases.

## Significance Statement

This study used wide-field, high-speed, and high-resolution VSD imaging (VSDI) to create a real-time functional map of intrahemispheric and interhemispheric connections in the PFC of mice. The PFC and ACC have critical roles in neuropsychiatric disorders, and the study found that neural signals within the ACC exhibit directional biases, which could affect interhemispheric functional connections. This finding could pave the way for more effective neuropsychiatric disorder treatments. The functional map created with VSDI is a potent tool for exploring functional connections in the brain and could provide valuable insights into how the brain processes information.

## Introduction

The prefrontal cortex (PFC; Laubach et al., 2018) is central to the integration of higher brain activities, such as working memory (Goldman-Rakic, 1995), mnemonic memory (Johnson et al., 2021), and social cognition (Apps et al., 2016). Accordingly, its disruption can cause schizophrenic and other neuropsychiatric phenotypes (Ghosal et al., 2017; Yan and Rein, 2022). In particular, the interhemispheric connection within the PFC through the corpus callosum (CC) plays a vital role in the pathology of these abnormalities (Aboitiz and Montiel, 2003; Tovar-Moll et al., 2007; Walker et al., 2012; Fenlon and Richards, 2015; Rovira and Geijo-Barrientos, 2016; Fenlon et al., 2021; Kilroy et al., 2022). The CC, particularly the anterior CC, participates in interhemispheric bilateral propagation of epileptic discharges (Musgrave and Gloor, 1980; Brodovskaya et al., 2022), and is, thus, a focus for the surgical treatment of epilepsy (Asadi-Pooya et al., 2008; Bullinger et al., 2022). However, the mechanism by which ipsilateral neural activity propagates to the CC and spreads to the contralateral hemisphere, especially in the frontal lobe, is not well understood.

Voltage-sensitive dye (VSD) imaging (VSDI) can directly visualize primary neuronal signals, including excitatory and inhibitory signals. Most functional imaging methods measure slow metabolic activity and secondary messenger (Ca^2+^) levels. However, real-time imaging techniques with high speed and high resolution have become necessary as interactions within and between cortical columns/hemispheres occur on a millisecond timescale. Therefore, tracking neuronal computations at the fundamental level of cortical columns in real time requires a spatial resolution of ∼100 μm and a temporal resolution of ∼1 ms (Buzsáki et al., 2012; Knöpfel and Song, 2019; Newton et al., 2021). Optical recording methods involving the external application of a dye (i.e., VSD) and high-speed and high-resolution capabilities will be advantageous in visualizing the broad correlations among brain areas from different angles *ex vivo* (Tanifuji et al., 1994; Iijima et al., 1996; de Curtis et al., 1999; Jin et al., 2002; Yuste, 2008). VSDI has shown high effectiveness in depicting various interactions among regions of the brain circuit and has been established as a quantitative and useful measurement tool (Tominaga et al., 2000, 2018, 2019) covering a wide area (Kajiwara et al., 2019; Kajiwara and Tominaga, 2021). Understanding how neural interactions are organized across multiple levels in the brain may provide a basis for fully elucidating higher brain functions in the PFC.

The main objective of this study was to create a functional map of the intrahemispheric and interhemispheric connections of the PFC in mice. To achieve this goal, we used the VSDI technique, which has been widely adopted in neuroscience research (Salzberg et al., 1973; Cohen et al., 1978; Cohen and Salzberg, 1978; Homma et al., 2009; Peterka et al., 2011; Tominaga et al., 2013; Roome and Kuhn, 2020), to visualize brain activity. Specifically, we used a specially designed wide-field imaging system equipped with high-speed, high-resolution capabilities and a large imager. With this system, we were able to record neural activity across the entire coronal slice of the PFC. The application of this system facilitated the construction of a detailed functional map of intrahemispheric and interhemispheric connections of the PFC, which is critical for understanding the neural mechanisms underlying neuropsychiatric disorders.

## Materials and Methods

### Animals

C57BL/6N male mice aged 4–8 weeks were obtained from a distributer (Japan SLC). All animal experiments were approved by the Animal Care and Use Committee of Tokushima Bunri University (#KP21-83–2, #KP22-83-2). All applicable international, national, and/or institutional guidelines for the care and use of animals were followed.

### Preparing brain slices and VSD staining

**Figure 1.**
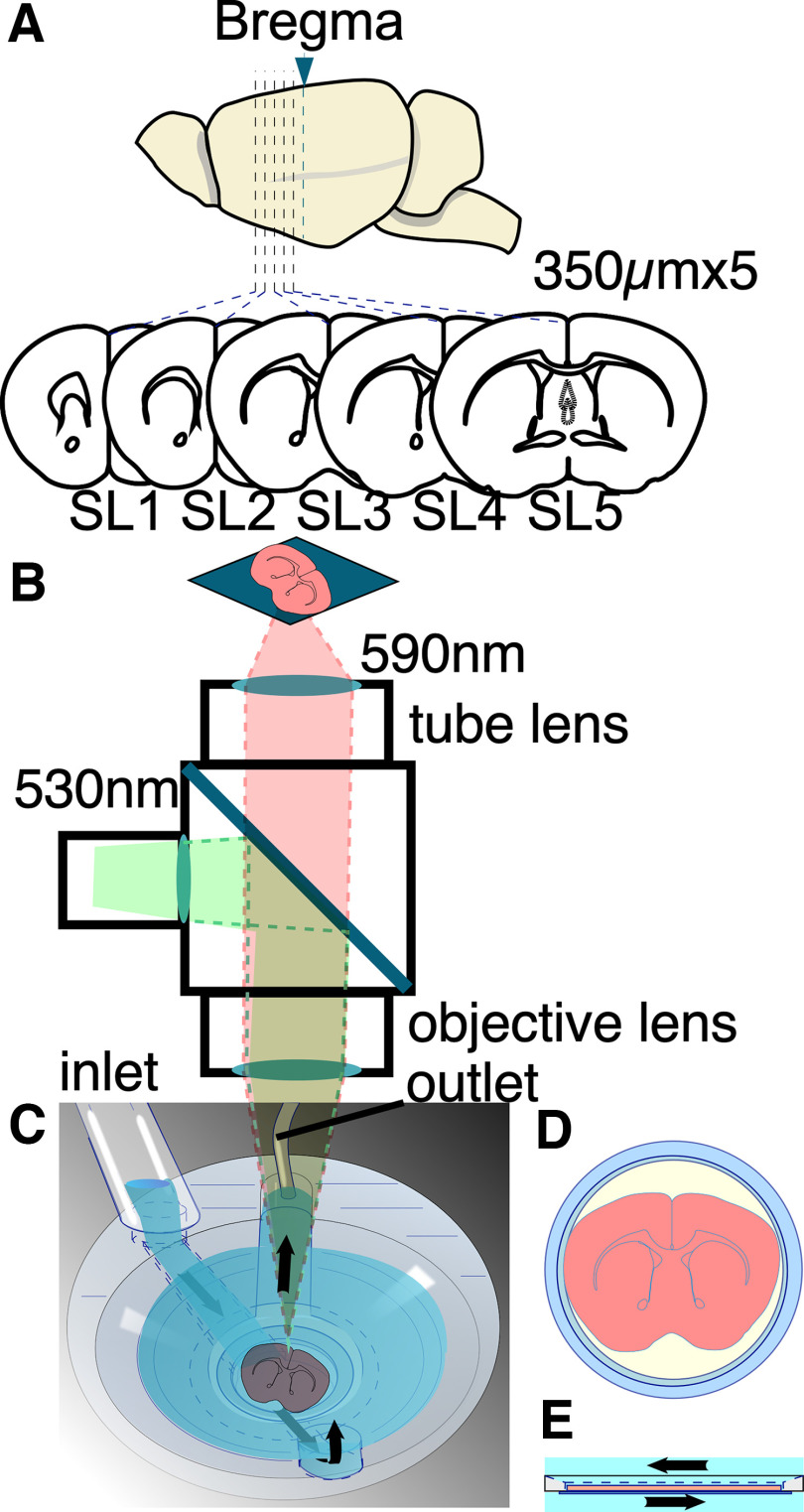
Schematics of slice preparation and experimental apparatus. ***A***, Schematic illustration of mouse brain slices. The slices (350 μm thickness) are continuously made from mouse brain. Each slice (5 slices per animal) is classified into SL1–SL5 depending on its morphology with reference to the brain atlas. SL1 refers to the slice collected 17.0 mm away from the bregma; SL2, 1.34 mm; SL3, 0.98 mm; SL4, 0.62 mm; and SL5, 0.26 mm. ***B***, Schematic illustration of the optical recording system. To briefly summarize the experimental setup, the excitation light first passed through a bandpass filter (530 ± 30 nm) and was then reflected by a dichroic mirror and directed toward the specimen. The resulting fluorescence was captured using a long-pass filter (>590 nm) and projected onto an imager. The optical system consisted of an objective lens and a tube lens with the same *F* value, resulting in a total magnification of one. ***C–E***, Recording chamber used for stabilizing the slice on a membrane filter (***D***) and for passing gas and fluid beneath the filter (***E***). The ACSF was delivered through the inlet and perfused from the bottom of the slice before being removed via the outlet.

Mice were anesthetized with isoflurane in a fume hood. The brain was then immediately resected and placed in cold artificial CSF (ACSF) solution containing the following (in mm): 124 NaCl, 2.5 KCl, 2 CaCl_2_, 2 MgSO_4_, 1.25 NaHPO_4_, 26 NaHCO_3_ and 10 glucose, pH 7.4, for 5 min. Thereafter, the PFC was sectioned into 350-μm-thick coronal slices using a vibrating slicer (VT-1000 or VT-1200; Leica Microsystems; Fig. 1*A*). Each slice was transferred into a special holder with a membrane filter (Tominaga et al., 2000, 2019) designed to keep the slices viable and maintain their order. The sections were then compared with the images on an atlas (Paxinos and Franklin, 2008) and labeled slice (SL)1–SL5 according to their distance from the bregma, 1.70, 1.34, 0.98, 0.62, and 0.26 mm (Fig. 1*A*).

Five slices were collected from each animal (*n =* 8). All sections were then transferred to a humid chamber containing ACSF solution and a continuous supply of 95% O_2_/5% CO_2_ mixed gas. The slices were incubated at 28°C for 25 min and stored at room temperature (22–26°C) for ∼10–15 min before VSD staining. After 40 min of incubation, each slice was stained with 110 μl of VSD solution for 20 min. The dye solution contained 0.2 mm Di-4-ANEPPS in 2.5% ethanol, 0.13% Cremophor EL, 1.17% distilled water, 48.1% fetal bovine serum, and 48.1% ACSF (Tominaga et al., 2000, 2013, 2019, 2023).

### Optical recording

Each slice was placed in the recording chamber with a continuous perfusion of oxygenated ACSF (bubbled with a 95%/5% O_2_/CO_2_ gas mixture) at a rate of 1 ml/min. To maintain cortical activity similar to that observed *in vivo,* all the data presented in this article were collected under perfusion of 1 μm SR95531 (gabazine; Tocris Bioscience). This approach has been previously used to suppress excessive inhibitory synaptic transmission that can occur because of differences in the slice condition compared with *in vivo* conditions, thereby preserving cortical activity (Iijima et al., 1996; Kajiwara et al., 2019). Epifluorescence optics with two identical lenses (×1 objective lens for a stereo microscope MZ series; catalog #10450028, Leica Microsystems) were used to visualize the slices—one for an objective and the other as a tube lens (Fig. 1*B*). The stained sections were illuminated with an excitation light from a stabilized LED light source (LEX2-LZ4; Brain Vision) passed through a filter (530 ± 30 nm). The amount of fluorescence generated by the stained section was passed through an emission filter (>590 nm) and projected onto a camera.

To capture a wide range of neural activity, we used a specially designed wide-field imaging system with high-speed, high-resolution capabilities and a large imager (MiCAM05, Brain Vision). This system allowed us to record neural activity in the entire coronal slice at a high frame rate (1 ms/frame unless otherwise stated) and a high spatial resolution (256 × 256 pixels). A microcapillary glass (outer diameter, 1.0 mm; inner diameter, 0.75 mm) filled with ACSF solution was used as the stimulating electrode. A ground electrode filled with 3 m KCl solution was used to avoid potential differences during the experiment. After the stimulation electrode was placed on the slice, the recording began. The stimulation frequency was set every 20 s for four sets for each slice. The data obtained were then analyzed using the BV analysis program (Brain Vision Analyzer, Brain Vision) and a custom application program on Igor Pro (vesions 8 and 9, WaveMetrics). For numerical and statistical evaluations, a specially designed macro within Igor Pro software was used. The study incorporated the multiple-comparison Tukey’s (HSD) test function within Igor Pro for all statistical analyses. Vector field analysis, which investigated neural activity propagation recorded via the VSD signal, was executed using the gradient function in the Python (version 3.9.16) Numpy package (version 1.23.5).

Electrical stimulation was provided with a glass electrode filled with ACSF (<1 MΩ, bipolar 40 V, 0.5 ms each) to nine different sites in the slice [motor cortex, anterior cingulate cortex (ACC), and CC; Fig. 1*D*] from a stimulator (ESTM-8, Brain Vision).

### Neuronal tracing

To label the callosal neurons, solid DiIC18(3) crystals (catalog #041-33423, FUJIFILM Wako Chemicals) were added to the ACC sections under microscope guidance. Briefly, DiI crystals were added to the cingulate cortex in the ipsilateral hemisphere using a glass micropipette with a sharp, elongated tip. To ensure delivery of the dye to the surface, the slices were gently poked with the pipette containing the DiI crystal. Given the slower diffusion of the dye in the cortex, the slices were maintained in an oxygenated medium (bubbled with a 95%/5% O_2_/CO_2_ gas mixture) for 5–6 h. Finally, the slices were fixed with 4% paraformaldehyde and incubated at room temperature for 1–2 d to further ensure complete diffusion of the dye. Slices with 4% fixative were first bathed in 10× PBS for 30 min and then analyzed.

### Laser confocal microscopic imaging

The stained sections were imaged using an LSM 510 META microscope (Zeiss). All images were captured using ACHROPLAN 40×/0.80 w with a frame size of 1024 × 1024. The fluorescence emitted by DiI 543 nm was visualized using a helium/neon laser with the following configurations: HFT 480/543 main beam splitter, NFT 545 (secondary dichroic beam splitter), and LP505 BP 565–615 (bandpass filter).

## Results

### Intrahemispheric and interhemispheric propagations

**Figure 2.**
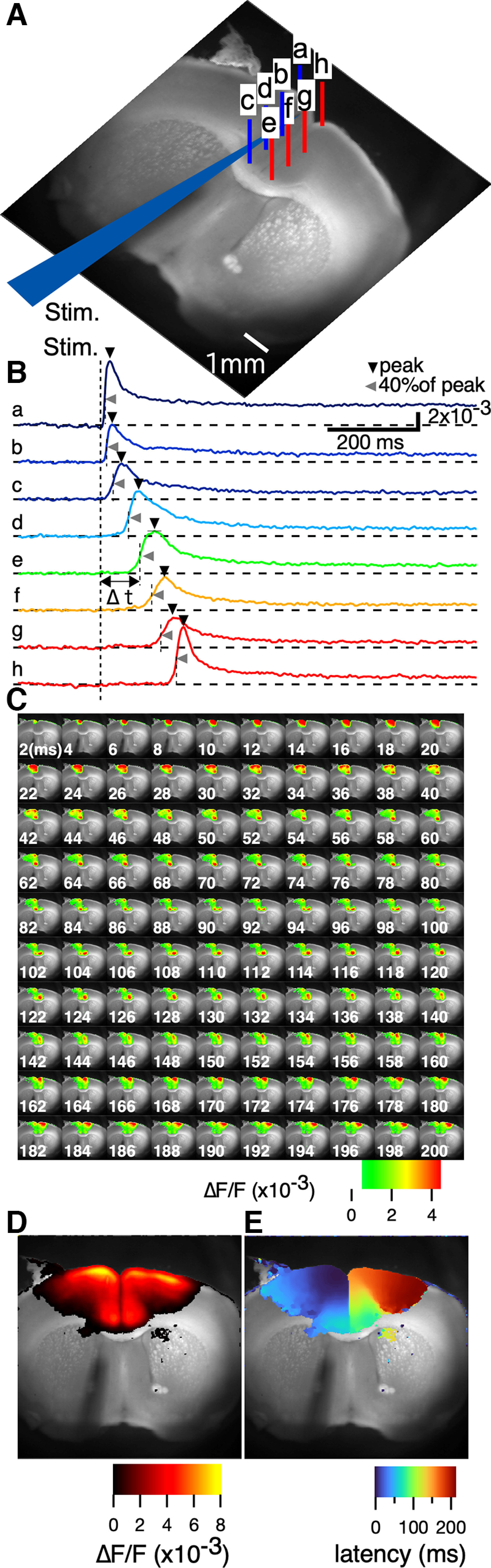
Contralateral spread of activity after electrical stimulation to the ACC. ***Aa–h***, Configuration of SL 3 (obtained 0.98 mm from the bregma) and the stimulation electrode. ***Ba–h***, Traces showing the optical signals at each pixel shown in ***Aa–h***. The vertical dotted line shows the timing of the stimulation (Stim.; 40 V, 300 μs bipolar). ***C***, Pseudocolored consecutive images of the optical signal at each time section (frame rate, 1 ms/frame). The numbers in the images indicate the time (ms) after the stimulation. ***D***, Color-coded projection of the peak values of each optical signal at each pixel in the field of view. ***E***, Color-coded projection map of the latency (Δt in ***B***; time to 40% of peak) to the initial response from stimulation time at each pixel in the field of view.

Electrical stimulation of the superficial layer (layer II/III) of the ACC [cingulate cortex area 1 (cg1)] in SL3 (0.98 mm from the bregma; Fig. 2*A*) elicited a transient depolarizing optical response at the site of stimulation (Fig. 2*Ba*). The optical signal at the pixel propagated to the medial side of the stimulated (ipsilateral) cortex (Fig. 2*Ba–d*, blue traces,). The propagation then spread across the interhemispheric connection to the contralateral cortex (i.e., other side of the cortex; Fig. 2*Be–h*, red). Figure 2*C* shows the propagation as the amplitude of the optical signal at each time slice (1 ms/frame) in pseudocolor in the ipsilateral and contralateral cortex. The septal-directed propagation along the ACC reached the distal end of cingulate cortex area 2 (cg2) within 39 ms (Fig. 2*C*). It then propagated to the contralateral side. The response appeared in the lower part of cg2 on the contralateral side within ∼49 ms. The wavefront of activity then moved to the dorsal side of the slice along with the ACC.

The propagation occurred as the wavefront of the activity appeared perpendicular to the layer structure of the cortex, indicating that the entire layer demonstrated consistent latency. For further analysis of functional activity mapping, we summarized the dynamic data in both the amplitude and time domains. The projection of the maximum peak, V_peak_ as visualized in Figure 2*Ba*, surpasses a threshold value of 0.5 × 10^−3^ and is displayed in Figure 2*D* in pseudocolor. The pattern of the activation amplitude map showed symmetry relative to the midline. The time that elapsed from the point of stimulation, marked by the dashed line t0 in Figure 2*B*, was determined when the signal amplitude achieved 40% of its peak, denoted as Δt in Figure 2*Bh*. This latency for each pixel is color represented in the latency map shown in Figure 2*E*, adhering to the same threshold applied in Figure 2*D*. The activity propagated from the stimulation site and crossed the hemispheric boundary. The amplitude map delineates the neuronal networks activated within the cortical tissue, whereas the latency map reveals the order of their activation.

### Neuronal activation on different stimulation

**Figure 3.**
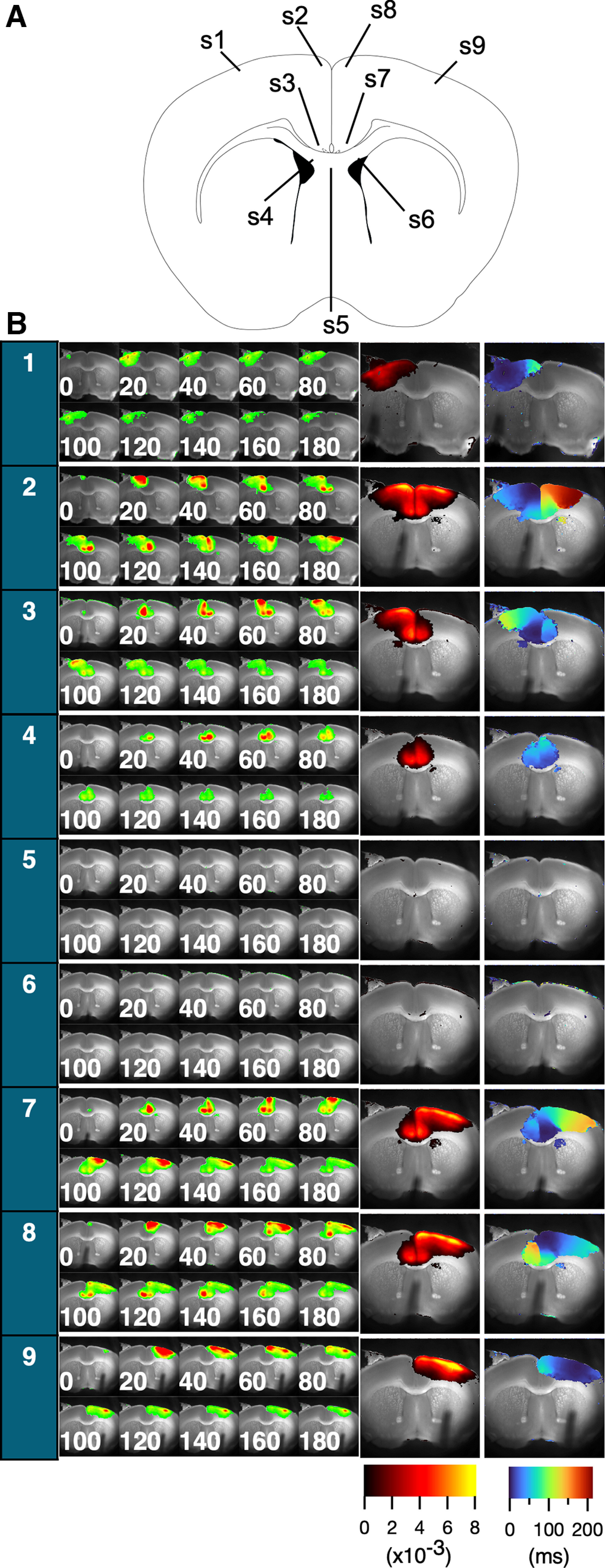
The figure displays consecutive images of the optical response (***A***), as well as projections of peak values and latencies for each stimulation site (***B***). Far left, Column corresponding to the site numbers (see above, Materials and Methods). The optical signal following stimulation is represented by 10 consecutive images, with each image numbered with a time point separated by 20 ms intervals and presented as a pseudocolor. Right, The two images show the projection of the peak values and latencies from the time of stimulation, presented in pseudocolor code as amplitude and latency maps, respectively. The amplitude maps show the amount of ΔF/F (×10^−3^), whereas the latency maps show the latency time in milliseconds, as indicated by the color bars (bottom right).

To determine whether the same neural circuit is consistently activated by stimulation applied to different sites, we characterized which area is activated (amplitude maps) and how these circuits respond (latency maps) in the cortex. To this end, we stimulated nine different sites [stimulation site (s)1-s9] in the slice [layer II/III of the motor cortex (s1, s9), cg1 and cg2 of ACC (s2, s8, s3, and s7), the site opposite cg2 (s4 and s6), and middle of CC (s5); Fig. 1*D*]. Figure 3*A* shows the activation pattern of each stimulation site (10 consecutive images every 20 ms from stimulation) and the projection map of amplitude and latency.

The amplitude maps revealed a similar pattern; however, in most cases (Fig. 3*B*, s1, s3, s4, s7, s8, and s9), the amplitude maps were within the broadest spread (Fig. 3*B*, s2). This suggested that the local activation of the neural circuit had a limited spreading capacity. Stimulation of both hemispheric cortices resulted in nearly identical activation patterns (s1 vs s9, s2 vs s8, and s3 vs s7). Activity in the ventral side of the ACC (cg2) was followed by activation of the lateral dorsal striatum. Stimulation of the white matter (S5 and S6) did not induce a clear response. Notably, white matter stimulations, although capable of inducing activation in adjacent cortical areas, generally necessitate a higher activation threshold than direct cortical stimulations. This might reflect inherent differences in the activation thresholds intrinsic to these respective regions.

### Mapping of the activity spread in different slices

**Figure 4.**
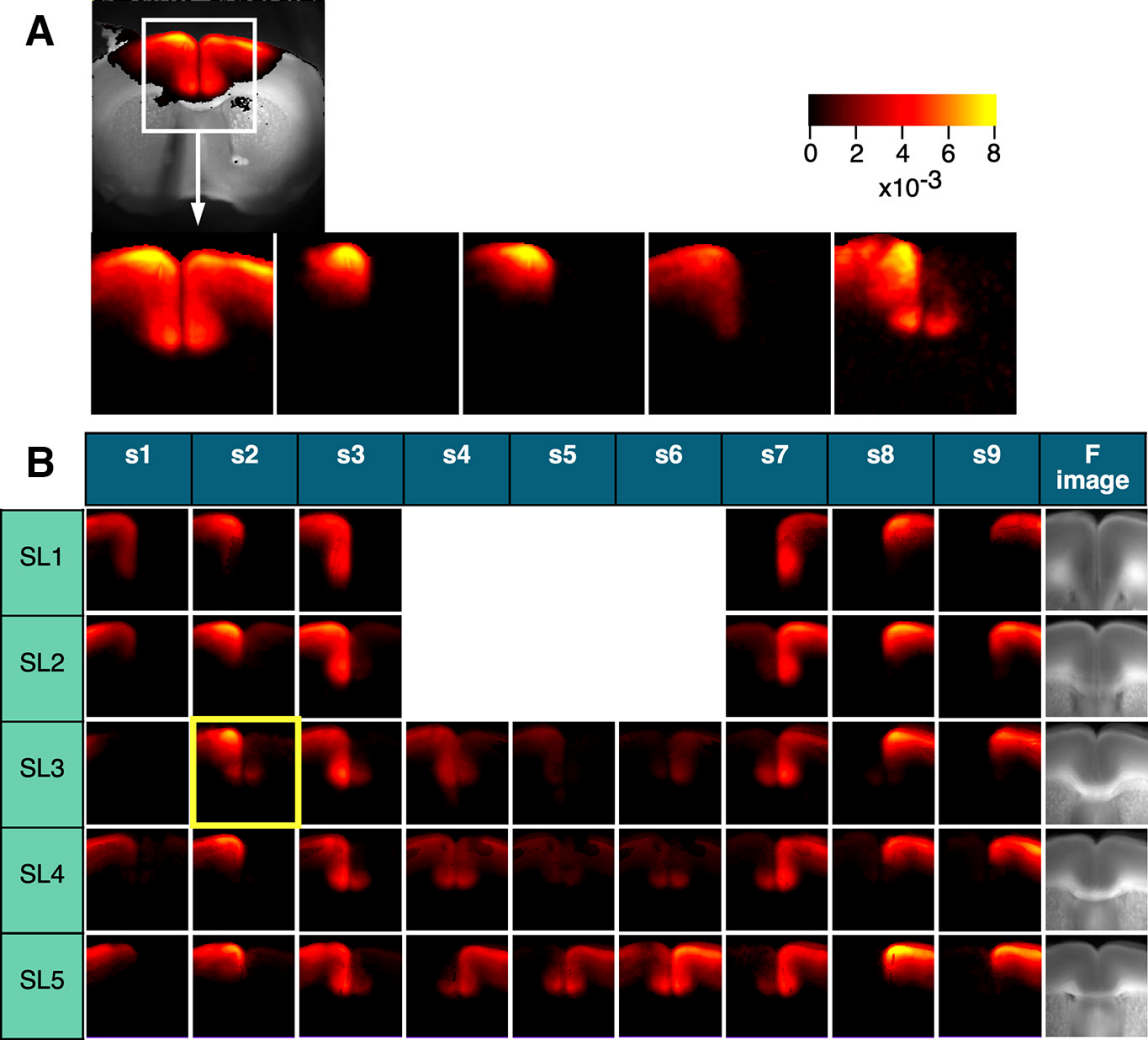
Map of the average peak-value projection across different slices. ***A***, The variation of the same response to S2 in five different SL3 slices (0.98 mm from the bregma). ***B***, Table of average peak-value projections in nine different stimuli (S1–S9) for five different slices (SL1–SL5). The averaged image highlighted in yellow (SL3/S2) is the average of five different responses shown in ***A***. The averaged images are generated after affine conversion and trimming to best fit the shape of the slice; *n =* 6–8 per image.

The amplitude and latency maps are essential for understanding the neuronal circuitry in the cortex as they provide information on the circuits recruited by local activation. By examining these maps, we can gain insights into the specific networks involved, which is critical for mapping and analyzing the complex neuronal circuits in the cortex. To create a functional map of the PFC, we constructed averaged images by selecting regions of interest that matched the morphology of different brain slices. This approach allowed us to identify patterns of neural activity across different areas of the PFC and generate a comprehensive functional map of this brain region. Figure 4*A* presents five amplitude maps obtained from a similar slice plane (SL3; 0.98 mm from the bregma) on the same surface stimulated at cg1 (S2). We created an averaged image from these five recordings (Fig. 4*B*, SL3/s2, highlighted in yellow) by cropping the region and applying affine convolution (rotation of the image). The averaged image on SL3/s2 represented data from five different animals. Figure 4*B* shows the averaged images over five different levels (SL1–SL5) in the nine stimulation sites (S1–S9). The far right images show the averaged fluorescence image from SL1 to SL5. The propagation pattern was similar between the left and right hemispheres. In the following section, we used all data from the same slice level to analyze the ipsilateral side (the stimulated side) on the left and the contralateral side on the right.

### Asymmetric propagation of the neuronal signal in the ACC

**Figure 5.**
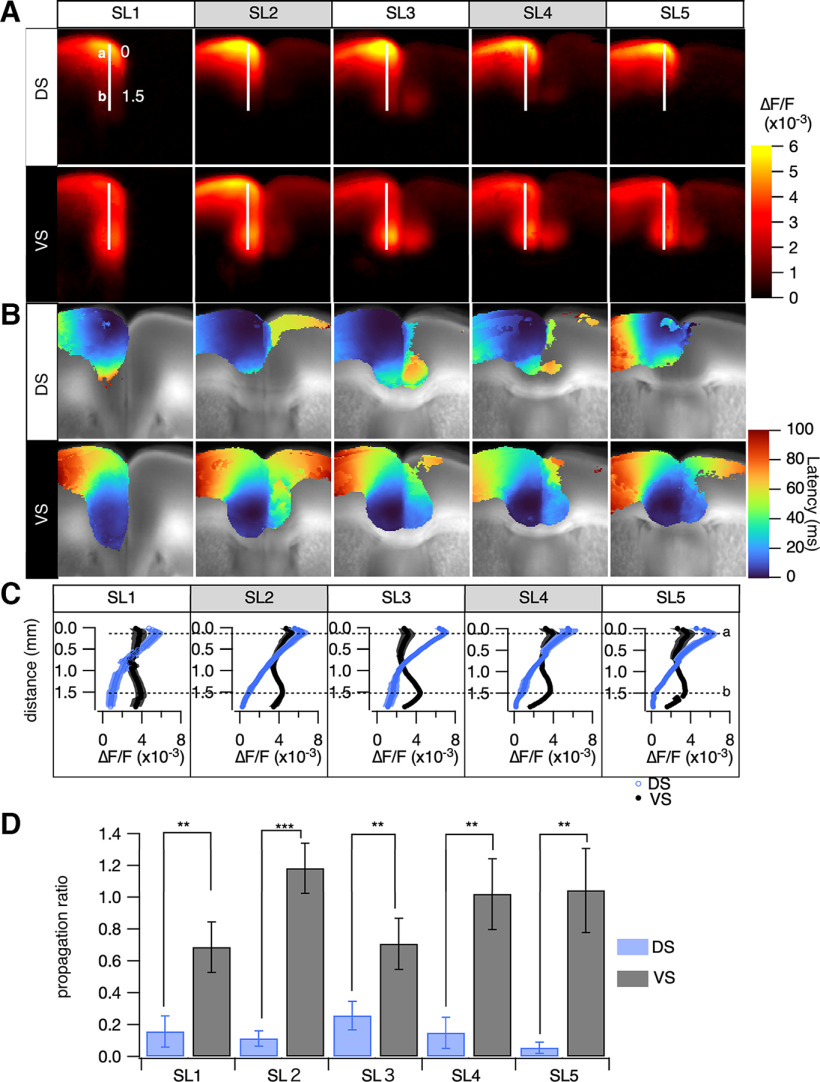
Analysis of uneven intrahemispheric neuronal propagation in the mPFC, comparing dorsal stimulation (DS) and ventral stimulation (VS) in layer II/III. ***A***, The averaged peak projection image, arranged to position the stimulation site (ipsilateral hemisphere) on the left and the contralateral hemisphere on the right. ***B***, The averaged latency projection, corresponding to ***A***, with latency defined as the time to reach 40% of peak amplitude from stimulation onset at each pixel. ***C***, Line profiles of peak projections (mean ± SEM; *n* = 6–8), depicting responses to DS (blue) and VS (black) along a specific line in ***A***, ranging from SL1 to SL5. Right, Dashed lines (***a***, ***b***) indicate locations nearest to the stimulation sites for DS and VS, respectively. ***D***, The propagation ratio, derived from the intensity of the optical signals (ΔF/F) at the dashed lines in ***C***. For DS, the ratio is ascertained by comparing the signal intensities at positions ***a***, ***b*** (dorsal to ventral), whereas for VS, the calculation is inverted from ***b*** to ***a*** (ventral to dorsal). The bar graph elucidates these ratios (mean ± SEM; *n* = 6-8), facilitating a comparative evaluation of neuronal propagation variability between different stimulation sites; **p < 0.03, ***p < 0.01.

The propagation patterns within the ACC are shown in Figure 5*A* (amplitude). Dorsal surface stimulation caused limited propagation to the ventral side of the ACC, whereas ventral stimulation caused more extensive propagation to the dorsal side of the ACC. Figure 5*A* shows images of the ACC, with white lines indicating a vertical line overlaid on the images. In Figure 5*B*, we present a plot of the amplitude profile along this vertical line, revealing how neural activity varies along this region of the ACC. The activity profiles induced by superficial stimulation rapidly decreased as the distance from the stimulation site increased. In contrast, deep stimulation produced a relatively flat amplitude profile along the line.

The propagation ratio is described in Figure 5*D*, showing disparities in amplitude ratios at points most distant from the stimulation sites between dorsal (blue) and ventral (gray) stimulations. Notably, across all sectors (SL1–SL5), the propagation ratio manifested significantly more robustly during ventral stimulation than dorsal stimulation. This highlights an intrinsic asymmetric propagation mechanism within the ACC. Our observations indicate a prevailing tendency for neural activity to propagate with enhanced intensity from the ventral to the dorsal regions of the medial PFC (mPFC). This inherent directional inclination in propagation mechanisms within the area appears to be intimately influenced by the prevailing pathways of information flow. Such observations furnish a nuanced understanding of the multifaceted roles of the mPFC in the processing and synthesis of information emanating from various brain regions. It further suggests the existence of specialized neuronal mechanisms within the mPFC, which are pivotal in facilitating these intricate processes.

### Cortical propagation velocity by area and direction

**Figure 6.**
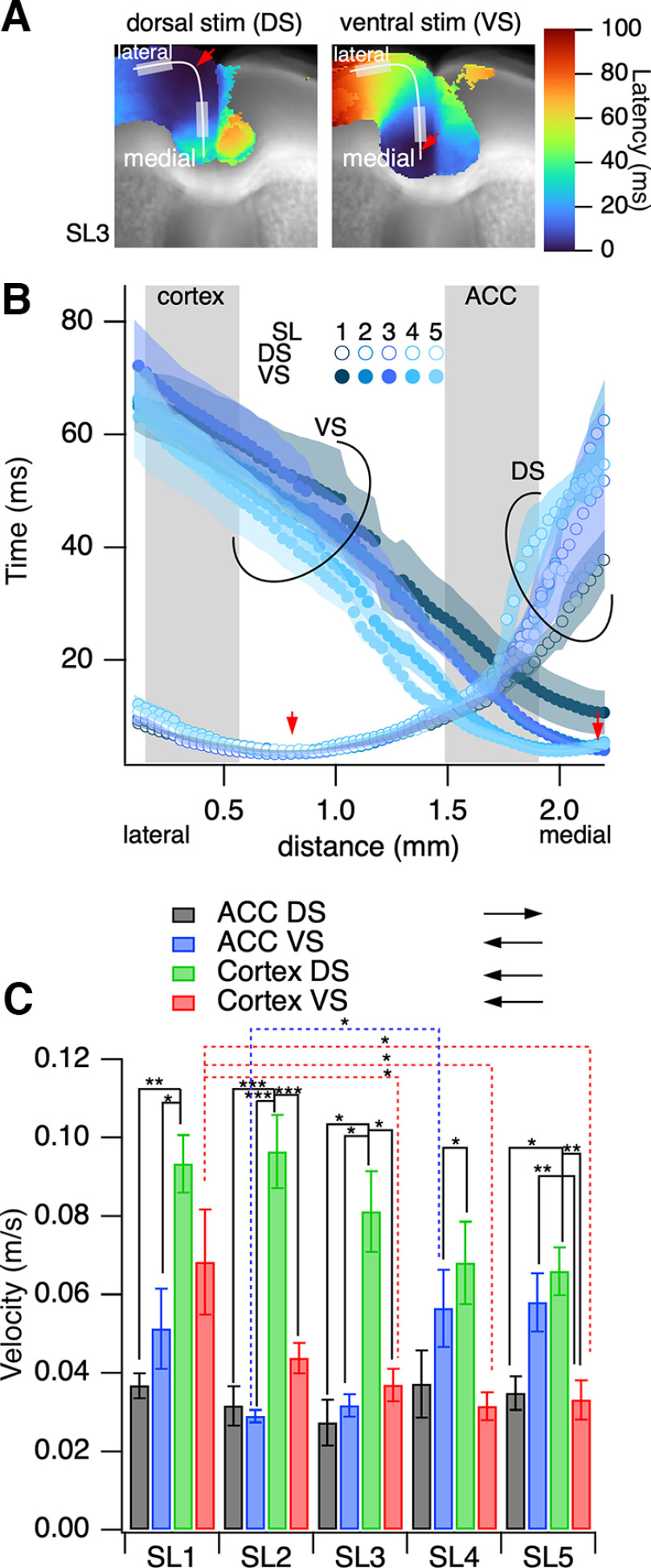
Analysis of intrahemispheric neuronal propagation in the mPFC on dorsal stimulation (DS) and ventral stimulation (VS) in layer II/III, to evaluate response latency. ***A***, The averaged latency projection image is summarized as having the stimulation site (ipsilateral hemisphere) on the left-hand side and the other (contralateral hemisphere) on the right. ***B***, The line profile of the latency along a line drawn on ***A*** (SL3) along layer II/III of the cortex on DS (open circle) and VS (solid circle). ***C***, The propagation velocity is calculated as the inverse of the slope of the profile at the lateral cortex (LC) and the ACC on DS and VS. The plots show the mean ± SEM, *n* = 8–12; *p < 0.05, **p < 0.03, ***p < 0.01.

Figure 6*A* shows the average projection map of the pseudocolor rise time in SL3, calculated similarly to that in Figure 5*B*. Figure 6*B* shows the line profiles of the latency along the cortex from the lateral side of the adjacent motor cortex to the medial ventral side toward the CC. The line profile for surface stimulation had minimum values of ∼0.7 mm (stimulation site) and increased in both directions. The line profile of deep stimulation had minimum values of ∼2 mm at the stimulation site, depending on the difference in the shape of the slices. The slope of the line profile tended to have a similar value but varied depending on the location and direction of propagation.

The latency slope was an inverse function of the propagation velocity at that location. Figure 6*C* shows the propagation velocity measured at the cortical area (0–0.5 mm) and within the medial PFC (distance, 1.5–2.0 mm). The propagation speed in the medial PFC was almost always slower than that in the cortex, whereas it tended to be faster in the caudal slices when stimulation was applied ventrally. The speed of propagation in the cortex was consistently significantly higher under dorsal stimulation than under stimulation in other areas. Meanwhile, when the propagation elicited in the deep dorsal side reached the cortex, the propagation tended to be slower, except for that between SL1 and SL3–SL5. The variation in propagation speed between cortical areas suggests that the mechanisms responsible for propagation differ based on cytoarchitecture. The target neural circuit for information transmission may differ depending on the stimulation direction (stimulation site).

### Interhemispheric propagation of neural signals in the medial cortex

**Figure 7.**
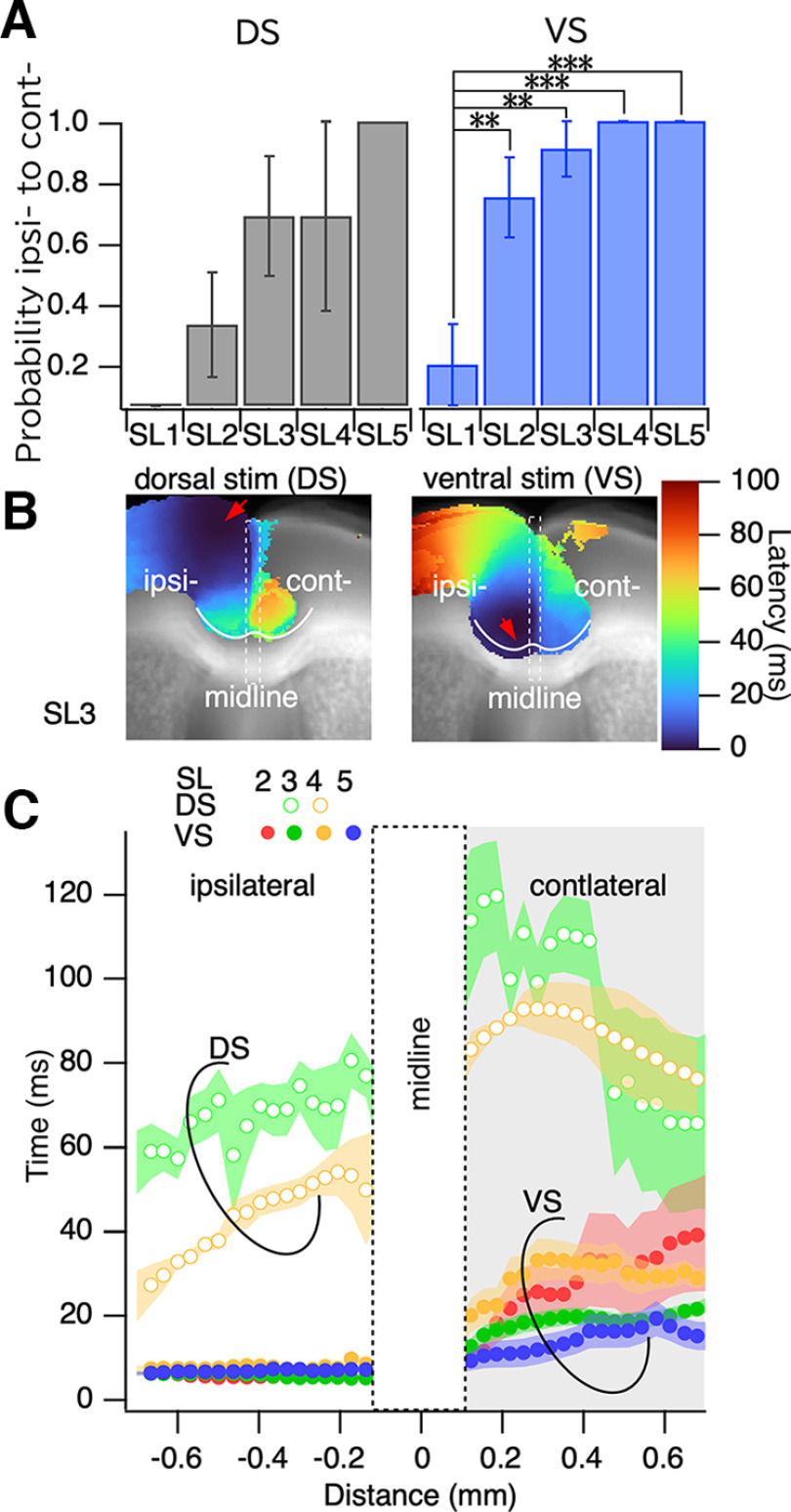
Interhemispheric connection. ***A***, Probability of the occurrence of interhemispheric connection from the ipsilateral cortex to the contralateral cortex. ***B***, Latency profile along the most ventral side of the medial prefrontal cortex drawn from the lateral end of the ipsilateral cortex to the contralateral cortex. ***C***, Profile of the latency plot along the line for dorsal stimulation (DS; open circles) and ventral stimulation (VS; solid circles); *n* = 8–12; **p < 0.03, ***p < 0.01.

Interhemispheric propagation differed in the slices. Figure 7*A* summarizes the probability of occurrence of interhemispheric propagation according to ipsilateral activity that could cause contralateral activity at the dorsal end of the PFC. The probability of interhemispheric propagation was significantly higher in the slices near the bregma, where the medial cortex was the ACC, than in the slices farther from the bregma.

Deep stimulation in the ipsilateral side showed a small increase over time. The minimum latency value in the contralateral side was 20 ms (SL2–SL5), which was similar to that in the ipsilateral side, and then increased to 30 ms (SL2–SL5). Meanwhile, under surface stimulation, the latency was decreased in the outer border (deeper side) of the ACC and then increased toward the septal border, lasting 30 ms (SL3, SL4). The rise time values in the contralateral side were lowest in the deep layer of the ACC. This suggested that the deep layer of the ACC may be a site of CC connection between hemispheres, at least for coherent neural signal propagation.

### The site of interhemispheric propagation

**Figure 8.**
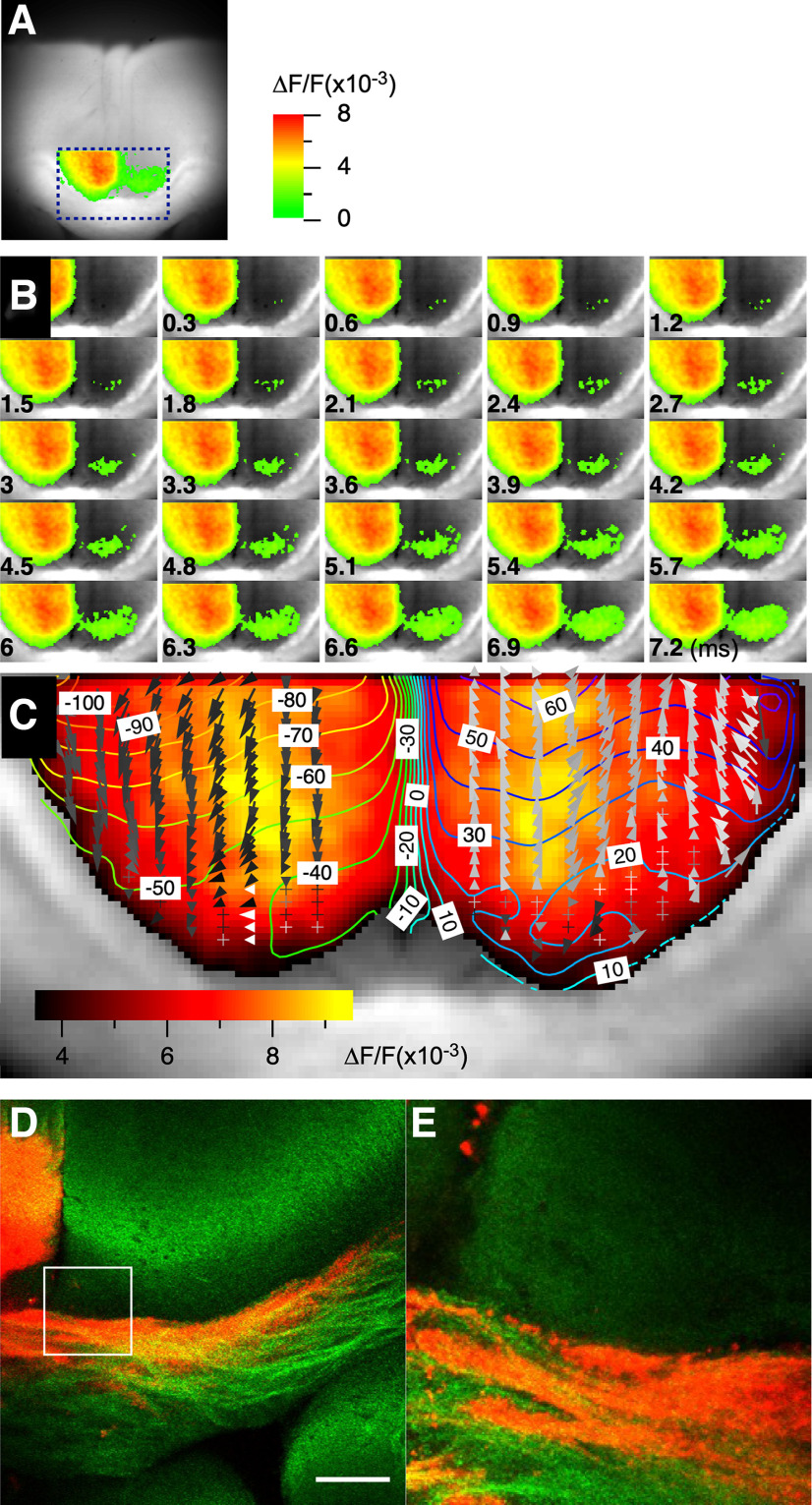
Site of interhemispheric propagation. ***A***, Location of the focal high-speed imaging (300 μs/frame) used to study interhemispheric propagation in the ACC. ***B***, Consecutive images taken at 300 μs intervals during interhemispheric propagation of neuronal activity in the ACC. ***C***, Amplitude map of the recorded activity superimposed with a contour plot and vector field representation of latency map, illustrating the direction and speed of the interhemispheric propagation. ***D***, ***E***, Representative images of DiI microdot staining in the ACC when the microdot was placed on the left side of the slice, showing the ipsilateral projection from the cingulate cortex and the corresponding interhemispheric callosal fiber projection to the contralateral side. Scale bar, 30 μm.

We addressed the site of interhemispheric connections in the ACC using high-speed focal imaging (300 μs/frame; Fig. 8*A*). Neuronal activation from the left side of the slice propagated interhemispherically, with activity first appearing in the deep layer of cg2 (Fig. 8*B*). The interhemispheric propagation occurred on the outer rim of the ACC and spread toward the ventral side of the brain. To better visualize the propagation, we created a contour plot superimposed with a vector field graph indicating the propagation direction (Fig. 8*C*).

To examine the importance of the CC in the transmission of neuronal activity, we used a microsurgical technique with a fine microcapillary (outer diameter, 1.0 mm; inner diameter, 0.5 mm). Our findings showed that lesions made close to the center of the callosum terminated activity, blocking the connectivity between the ipsilateral and contralateral hemisphere of the ACC. This observation supports the speculation that the deep layer of cg2 serves as the connection site where the callosal fiber conveys the projection of neural activity from one side of the hemisphere to the other.

To further elucidate this perspective, we used the lipophilic neuronal tracer DiI crystal in our investigation. After applying the DiI crystal to the ipsilateral cingulate cortex, we observed that the labeled projections extended through the CC, terminating at the bottom of layer VI of cg2 on the contralateral side without further invasion into the contralateral structures (Fig. 8*D*,*E*). These findings highlight the pivotal role the deep layers of cg2 and callosal fiber projections play in determining the specific sites and functional connectivity associated with interhemispheric propagation in the ACC.

## Discussion

### Main findings

This study used VSDI to create a real-time functional map of intrahemispheric and interhemispheric connections in the PFC of mice. The ACC exhibited directional biases in neural signaling that could have an impact on interhemispheric functional connections. Specifically, differences in the propagation pattern among ACC areas were observed that affected interhemispheric propagation in the ACC. Moreover, we observed that the interhemispheric activity propagation within the ACC was particularly prominent in a specific region, cg2. This observation is remarkable given that the CC typically facilitates more widespread homotopic connections between the hemispheres (De León Reyes et al., 2020). These findings provide insights into the mechanisms responsible for neural signal propagation in the ACC and suggest potential targets for treating neuropsychiatric disorders. Furthermore, this study demonstrates the power of VSDI in investigating brain function and highlights the importance of exploring functional connections in the brain.

Here, a functional activity map of the PFC was generated using VSDI. Given that the anatomic definition of the rodent PFC is controversial (Laubach et al., 2018; van Heukelum et al., 2020), this study applied the nomenclature of Paxinos and Franklin (2008). Results revealed activity in the motor cortex (M1 and M2), ACC (cg1 and cg2), prelimbic and infralimbic cortex, and dorsal peduncular cortex. Additionally, evidence of activity was detected in parts of the dorsal lateral striatum (Fig. 2) and other cortical areas, particularly on the frontal side (mostly in SL1). These comprehensive data regarding the neural circuitry of the PFC were obtained because of the large field of view of the VSDI system, high spatial resolution (30 μm per pixel), and high frame rate (1 ms/frame). These conditions are typically challenging to achieve as they can easily lead to a reduced photon number at the imaging device, resulting in a poor signal-to-noise ratio.

In our study, we administered an electrical stimulation of ±40 V, exerted biphasically for 500 μs each. This level of stimulus strength was chosen based on its ability to elicit a maximal response in the CA1 area of mouse hippocampal slices, as supported by previous findings (Tominaga et al., 2019; Utsumi et al., 2023). Importantly, minor variations in the stimulation strength at this level had a negligible effect on the propagation pattern of activity within the cortical slices.

Within the mPFC and the interhemispheric region, the mode of propagation differed according to the propagation direction. Specifically, the neural signal propagating from the ventral to the dorsal cortical area (brain surface) had a greater probability of propagating from the CC to the dorsal cortical area. In contrast, activity from the cortex was strongly attenuated in the mPFC (Fig. 5). The mPFC has been implicated in several neuropsychiatric disorders. For instance, Strakowski et al. (2005, 2012) reported that emotional dysregulation in bipolar disorder results from the inability of the PFC to modulate anterior limbic structures such as the amygdala. Meanwhile, Ragland et al. (2007) concluded that the inefficiency in cognitive information processing in schizophrenia results from PFC dysfunction. Gilbert et al. (2006) also suggest that mPFC alterations contribute to impaired reality monitoring in schizophrenia (Gilbert et al., 2006). In addition, the ACC plays a pivotal role in pain-induced depression (Barthas et al., 2015), pain perception and chronic pain (Koga et al., 2015; Guo et al., 2022), and spinal sensory transmission (Chen et al., 2018). The present findings provide a functional map for investigating pathologic modifications in the PFC.

### Population propagation of neural activity under weak GABAergic blockade

The propagation of neural activity in layers II/III and intrahemispheric connections under GABAergic blockade has been extensively studied using electrophysiological methods (Walker et al., 2012; Rovira and Geijo-Barrientos, 2016; Robles et al., 2020; Domínguez-Sala et al., 2022). However, the present study did not detect any oscillatory activity under these conditions, possibly because of the weak blockade of GABAergic systems. Nevertheless, the propagation of the initial inward discharge in the field potential recordings (Rovira and Geijo-Barrientos, 2016) was similar to that observed with VSD imaging; that is, the propagation speed of 30–40 mm/s was relatively the same as that observed under GABAergic blockade. VSDI allowed us to visualize the mechanisms by which the depolarizing signal propagated through the ACC and activated all layers almost simultaneously.

The coherent neural propagation observed in the PFC at 30–40 mm/s is common in other cortical areas, including the entorhinal, perirhinal, and visual cortices (Iijima et al., 1996; Yoshimura et al., 2016; Kajiwara et al., 2019; Kajiwara and Tominaga, 2021; Fukuda et al., 2023). Detailed modeling has previously shown that PFC networks with strong feedback inhibition exhibit resonance (Sherfey et al., 2018). Increasing experimental evidence also shows millisecond fidelity and temporal reliability (Compte et al., 2000; Gollisch and Meister, 2008). With millisecond fidelity, precisely synchronized action potentials can propagate within a model of cortical network activity that mimics many of the characteristics of biological systems. This model demonstrates how time intervals and periodicity of operation can be determined by simulating synaptic learning in a neural circuit model based on neural connections (Durstewitz et al., 2000; He, 2019). Using a model of cortical network activity, Diesmann et al. (1999) showed that precisely synchronized action potentials can propagate with millisecond accuracy.

The cingulate cortex has been found to exhibit neural connectivity with motor areas on the lateral and medial surface of the brain as well as with the prefrontal cortex (Nauta, 1972; Vogt and Pandya, 1987; Koski and Paus, 2000). Neurons that facilitate interhemispheric connections project to the opposite cortex, particularly to the homotopic region of the brain (Tovar-Moll et al., 2007; Fame et al., 2011; Fenlon and Richards, 2015; De León Reyes et al., 2020; Szczupak et al., 2023).

The optical signal in this study did not show such direct activation of the homotopic region (Fig. 7). Rather, the optical signal propagated to the contralateral hemisphere at CG2 facing the CC. The results showed that microsurgery of the CC on the lateral side did not disrupt interhemispheric interaction, but resection in the middle of the CC disrupted propagation (Fig. 8). Although the initial stimulation was delivered to L2/3 cg1 and cg2, the neuronal activity captured by the VSD signal activated sequential propagation within the ACC and interhemispheric propagation to cg2. Although it is unclear whether this type of activity propagation carries physiological information, correlations of spikes from multiple neurons may have essential functions (Panzeri et al., 2022).

### Optical recording of cortical activity

Synchronized activity among cortical neurons is critical for normal brain function and allows the integration of information. Disruptions in this synchronized activity have been linked to various conditions, including epilepsy, schizophrenia, and Alzheimer’s disease (Singer, 1993; McNamara, 1994; Pinto et al., 2005; Takahashi et al., 2015; Muller et al., 2018). Previous research has emphasized the importance of synchronous activity for working memory and cognitive deficits in schizophrenia (Takahashi et al., 2015; Muller et al., 2018). It is measured with various imaging techniques, including genetically encoded Ca indicators (Huang et al., 2010; Rynes et al., 2021) and genetically encoded voltage indicators (Knöpfel, 2012; Knöpfel and Song, 2019; Rhee et al., 2021). VSDI is also a reliable tool for visualizing these synchronizing activities in the cortex (Tanifuji et al., 1994; Iijima et al., 1996; de Curtis et al., 1999; Jin et al., 2002; Yuste, 2008; Fujieda et al., 2015; Yoshimura et al., 2016; Kajiwara et al., 2019; Kajiwara and Tominaga, 2021; Newton et al., 2021; Palkar et al., 2023). The large wide-field bright optics and the special chamber system (Tominaga et al., 2000, 2023), allowing stable fixation of the slices, enable characterization of large-scale cortical activity. The stable long-term recording also enables the collection of a large volume of data (Tominaga et al., 2019).

One potential limitation of this study is that it used electrical stimulation to recruit neural activity, which can activate multiple elements of the neural circuitry. As such, it can be difficult to determine which specific neural pathways are activated and how this relates to the observed activity. This issue could be addressed using optogenetics, which allows for more precise control of neural activity by selectively activating specific populations of neurons with light (Yizhar et al., 2011). However, it should be noted that the combination of voltage imaging and optogenetics can add additional complexity to the study. For example, optogenetics can alter the characteristics of neural activity in ways that may not be fully understood or accounted for in the analysis.

Nevertheless, this study provides important insights into the mechanisms of neural propagation in the prefrontal cortex and the potential role of directional biases in interhemispheric communication. The findings of the study have implications in understanding the neural basis of neuropsychiatric disorders and could inform the development of more targeted interventions. Future studies could explore the use of optogenetics to overcome the limitations of electrical stimulation and to provide more precise control over neural activity in the prefrontal cortex and other brain regions.

## Synthesis

Reviewing Editor: Arvind Kumar, KTH Royal Institute of Technology Decisions are customarily a result of the Reviewing Editor and the peer reviewers coming together and discussing their recommendations until a consensus is reached. When revisions are invited, a fact-based synthesis statement explaining their decision and outlining what is needed to prepare a revision will be listed below. The following reviewer(s) agreed to reveal their identity: Thomas Knopfel. Note: If this manuscript was transferred from JNeurosci and a decision was made to accept the manuscript without peer review, a brief statement to this effect will instead be what is listed below.

Synthesis

Authors have characterized functional connectivity among frontal cortical areas using VSDI and electrical stimulation in mouse cortical slices. While the methodology may not be new, authors here present new data which gives some insights into the functional connectivity among frontal areas e.g. asymmetric functional connectivity between cg1 and cg2. The manuscript was reviewed by two reviewers. While both think that there is original data and interesting findings, the manuscript needs a major revision. The major concerns are:

Methods are rather poorly described. Even something as central to the work as the ‘propagation ratio’ and ‘activation’ are not properly defined.

There is no description of statistics. So when methods are missing or obscure and statistical tests are not described it becomes very difficult to accept the results.

English is rather poor. It would be necessary that a native speaker edits the manuscript

The two reviewers have made clear recommendations in their respective report. We invite you to revise the manuscript according to the reviewers’ comments and provide a point-by-point reply.

Reviewer #1

The authors used voltage imaging to assess the in vitro functional connectivity between prefrontal cortex (PPC) and anterior cingulate cortex (ACC) in mice. These structures have, in humans, critical roles in neuropsychiatric disorders and mouse models are established to study the circuit dynamics underlying basic PFC/ACC functions and dysfunctions.

The main merit of this study lies in the solid observational data. I am less enthusiastic about the presentation and insights offered in the discussion. I sense that defence/praise of the methodology is pre-emptive to criticism but, to my take, does not improve the quality of the paper.

The level of English language is sufficient to mostly comprehend everything but there is space for editing towards a more accessible language.

Specific comments:

The authors use at places the word “disc” for “brain slice”. This is a bit unconventional and may lead to confusion. If the term “brain slice” is not appropriate, an explanation of why would be helpful.

How is latency defined and measured?

How was “ratio of propagation” defined and measured?

Was there any spontaneous [spreading] activity in these preparations observed? This would be expected as inhibition was supressed by gabazine.

Figure 2C: Please include (selected) frames up to 200 ms to cover the range for which responses are shown in 2B and 2D&E

Line 331 ff: The following sentence needs to be reworded/expanded: “Moreover, interhemispheric connections occurred in a specific area of the ACC, cg2, which is not typical as the CC commonly connects the two hemispheres”. Do interhemispheric connections originate in or do they confine to cg2?

Line 345 ff: “... extended recording time (1 ms/frame) ...”. Does not make sense. Do you speak about frame interval (rate) or trace duration?

Line 369: What is meant with “initial inward discharge”.

Line 418 ff. “This wide-field imaging provides advantages to VSD staining and makes it more beneficial than the virus expression dependent expression analysis in GECI and GEVI, aside from the use of knock-in animals”. This statement makes little sense to me: (i) the optical approach that is employed here is not limited to one type of indicator. (ii) does “aside” mean “except”, and if so, why does this make “VSDs” beneficial?

Reviewer #2

Gusain et al. characterized functional connectivity among bilateral frontal cortical areas using VSDI and electrical stimulation in mouse cortical slices. Notable findings were bilateral cortical activation elicited by direct white matter stimulation, and asymmetric functional connectivity between cg1 and cg2. These findings potentially provide important implications for the mechanism of activity propagation in epilepsy.

While the objective and the experimental approach of the study are reasonable, it was difficult to evaluate the credibility of the present findings because of the lack of description about some of the key analyses. This manuscript also lacks statistical assessment of the findings. Although some bar graphs have indications of statistical significance, I could not find any description of statistics (name of statistical test, p value, n etc.). I think this paper needs to be substantially improved in order to undergo proper peer review.

Major points

1) Please add a description of statistics.

I could not find any description of statistics. Without statistics, it is not appropriate to claim that the results were “significant” (e.g. lines285 and 295). Some bar graphs contain asterisks that likely indicate statistical significance. Please describe the statistical testing (p values, statistics, n etc).

2) How did the authors define propagation ratio and activation?

In Figure 5D, authors use propagation ratio to quantify the difference between surface vs deep stimulation. I could not find a description of the method. Also, I could not find the definition of the activation. How did the authors define whether some pixels were activated or not activated? These points are essential for assessing the credibility of the main findings.

3) How was the strength of electrical stimulation determined?

The results of functional connectivity should depend on the strength of stimulation. Ideally, the authors could test multiple stimulation strengths and characterize/interpret the functional connectivity as a function of stimulation strength. If this is not possible, the authors should add a discussion to validate their choice of stimulus strength.

Minor points

Line 50: “256 x 256 pixels” ?

Line 111-112: I think calling VSDI a “cutting edge” technique is a bit misleading. VSDI has been around for more than 20 years.

Lines140-146: I am not an expert of VSD imaging, but I found this VSD (Di-4 ANEPPS solution?) different from commonly used VSDs (e.g. RH1691 by Grinvald’s group). It would be helpful for readers if you cite previous publication(s) that characterize the Di-4 ANEPPS solution for VSDI.

Lines230-231 states that stimulation of white matter (S4,S5,S6) did not produce a clear response. But, I think S4 in Fig3B shows a clear response. Please explain.

Figure 3B: Black numbers on blue background are very difficult to see. Please change the color.

Figure 4A shows substantial across animal variability. Please explain possible reasons.

Figure 4 lacks a colorbar indicating the activity level.

Figure 7A lacks indication of statistical significance, but the main text states significant difference across slices. Which one is true?

Figure 8D,E: Where is the “aberrant callosal projection”? Please indicate by arrows.

Also, is Figure8E a magnified view of the white rectangle in Figure 8D? They do not seem to match (Fig8E has square shape).

## Author Response

Synthesis of Reviews:

Synthesis Statement for Author (Required):

Synthesis

Authors have characterized functional connectivity among frontal cortical areas using VSDI and electrical stimulation in mouse cortical slices. While the methodology may not be new, authors here present new data which gives some insights into the functional connectivity among frontal areas e.g. asymmetric functional connectivity between cg1 and cg2. The manuscript was reviewed by two reviewers. While both think that there is original data and interesting findings, the manuscript needs a major revision. The major concerns are:

Methods are rather poorly described. Even something as central to the work as the ‘propagation ratio’ and ‘activation’ are not properly defined.

There is no description of statistics. So when methods are missing or obscure and statistical tests are not described it becomes very difficult to accept the results.

English is rather poor. It would be necessary that a native speaker edits the manuscript

The two reviewers have made clear recommendations in their respective report. We invite you to revise the manuscript according to the reviewers’ comments and provide a point-by-point reply.

Reviewer #1

The authors used voltage imaging to assess the in vitro functional connectivity between prefrontal cortex (PPC) and anterior cingulate cortex (ACC) in mice. These structures have, in humans, critical roles in neuropsychiatric disorders and mouse models are established to study the circuit dynamics underlying basic PFC/ACC functions and dysfunctions.

The main merit of this study lies in the solid observational data. I am less enthusiastic about the presentation and insights offered in the discussion. I sense that defence/praise of the methodology is pre-emptive to criticism but, to my take, does not improve the quality of the paper.

The level of English language is sufficient to mostly comprehend everything but there is space for editing towards a more accessible language.

Specific comments:

R: The authors use at places the word “disc” for “brain slice”. This is a bit unconventional and may lead to confusion. If the term “brain slice” is not appropriate, an explanation of why would be helpful.

A: We appreciate your observation and agree that clarity and consistency in terminology are crucial. We have revised the manuscript to replace the term “disc” with “brain slice” to avoid any confusion and ensure that the terminology aligns with conventional usage in the field. Thank you for bringing this to our attention.

R:How is latency defined and measured?

A: Thank you for your question. Latency in our study refers to the time interval between the application of the stimulus and the moment when a certain proportion (40%) of the peak amplitude response is reached at a designated pixel. This methodology allows us to have a standardized measurement across different slices and stimulation sites, enabling a more consistent and reliable comparison of response times. We have clarified this definition and measurement method in the text and updated Figure 2 to better represent this information. We appreciate your attention to this detail.

R: How was “ratio of propagation” defined and measured?

A: Thank you for your question. The “ratio of propagation” is a metric we introduced to quantify the disparity in the amplitude of neural responses across different regions of the ACC. Specifically, it is calculated as the ratio of the response amplitude at the most dorsal side to that at the ventral side of the ACC. A ratio closer to 1 indicates that the response size remained consistent up to the end of the ACC.

To improve clarity and understanding, we have revised the manuscript to include a more detailed explanation of how the “ratio of propagation” is defined and measured.

In the updated text:

The propagation ratio is described in Figure 5D, showing disparities in amplitude ratios at points most distant from the stimulation sites between dorsal (blue) and ventral (grey) stimulations. Notably, across all sectors (SL1 to SL5), the propagation ratio manifested significantly more robustly during ventral stimulation than dorsal stimulation. This highlights an intrinsic asymmetric propagation mechanism within the ACC. Our observations indicate a prevailing tendency for neural activity to propagate with enhanced intensity from the ventral to the dorsal regions of the mPFC. This inherent directional inclination in propagation mechanisms within the area appears to be intimately influenced by the prevailing pathways of information flow. Such observations furnish a nuanced understanding of the multifaceted roles of the mPFC in the processing and synthesis of information from various brain regions. It further suggests the existence of specialized neuronal mechanisms within the mPFC which are pivotal in facilitating these intricate processes.

R: Was there any spontaneous [spreading] activity in these preparations observed? This would be expected as inhibition was supressed by gabazine.

A: Thank you for your insightful question. Under the conditions of our experiments, where 1 μM of SR95531 (gabazine) was used, we did not observe spontaneous spreading activity in the preparations. This was the case in the series of experiments we conducted.

Notably, in most frontal preparations, the neural activity we recorded tended to be prolonged over the recording period. This prolongation could sometimes be followed by a spontaneous or repetitive spread of activity. Furthermore, it is worth mentioning that applying a higher dose of gabazine could indeed induce spontaneous or repetitive activities in the slices, but such conditions were outside the scope of the observations reported in this study.

In reflection of your valuable input, we have adjusted the details in the manuscript to ensure that the conditions under which the experiments were conducted and the observations made are communicated with the utmost clarity.

R:Figure 2C: Please include (selected) frames up to 200 ms to cover the range for which responses are shown in 2B and 2D&E

A: We appreciate your attention to detail and the thoughtful suggestion. In response, we have updated Figure 2C to include selected frames up to 200 ms. This modification ensures that the figure comprehensively covers the range for which responses are illustrated in Figures 2B, 2D, and 2E, thereby providing a more coherent and complete visual representation of the data. We trust that this revision enhances the clarity and comprehensiveness of the presented results. Thank you for helping improve the quality of our presentation.

R:Line 331 ff: The following sentence needs to be reworded/expanded: “Moreover, interhemispheric connections occurred in a specific area of the ACC, cg2, which is not typical as the CC commonly connects the two hemispheres”. Do interhemispheric connections originate in or do they confine to cg2?

A: Thank you for bringing this to our attention. We apologize for the confusion. We have revised the sentence to better convey that the interhemispheric activity propagation appears to be notably concentrated within a distinct area of the ACC, referred to as cg2.

Revised text:

“Moreover, the interhemispheric activity propagation within the ACC was particularly prominent in a specific region: cg2. This observation is remarkable given that the CC typically facilitates more widespread homotopic connections between the hemispheres.”

We hope this clarification effectively addresses your query and enhances the clarity of our findings.

R:　Line 345 ff: “... extended recording time (1 ms/frame) ...”. Does not make sense. Do you speak about frame interval (rate) or trace duration?

A: We appreciate your careful reading and valuable feedback. We apologize for any misunderstanding caused by the terminology used. To clarify, “extended recording time (1 ms/frame)” refers to the frame rate of our recordings, meaning that our system captured one frame every millisecond. This allowed us to achieve detailed temporal resolution in capturing the dynamic neural activities. We have ensured that this is more clearly articulated in the revised text. Thank you for bringing this to our attention, and we hope this amendment improves the clarity of our article.

Revised text:

” This comprehensive data regarding the neural circuitry of the PFC was obtained due to the VSDI system’s large field of view, high spatial resolution (30 μm/pixel), and high frame rate (1 ms/frame). These conditions are typically challenging to achieve as they can easily lead to a reduced photon number at the imaging device, resulting in a poor signal-to-noise ratio.”

R: Line 369: What is meant with “initial inward discharge”.

A: We appreciate your question. The phrase “initial inward discharge” indeed refers to the phenomena observed in field potential recordings, as cited early in the manuscript. This term is utilized to describe the immediate neuronal response characterized by a distinct downward deflection in the field potential recordings post-stimulation. We apologize for any ambiguity in the text, and we are thankful for your input, which has allowed us to clarify this terminology in the manuscript.

R: Line 418 ff. “This wide-field imaging provides advantages to VSD staining and makes it more beneficial than the virus expression dependent expression analysis in GECI and GEVI, aside from the use of knock-in animals”. This statement makes little sense to me: (i) the optical approach that is employed here is not limited to one type of indicator. (ii) does “aside” mean “except”, and if so, why does this make “VSDs” beneficial?

A: We apologize for any confusion caused by our statement, and we appreciate the reviewer’s insightful comments. You are correct; the wide-field imaging approach employed in this study is versatile and not confined to voltage-sensitive dye (VSD) staining, as it can be applied with various indicators. Regarding the comparison made between VSD staining and the virus expression-dependent expression analysis in genetically encoded calcium and voltage indicators (GECI and GEVI, respectively), we acknowledge that the phrasing may have been unclear. Given your feedback and upon further reflection, we have decided to remove this sentence from the manuscript to maintain focus on the findings and methodology directly related to our study.

Reviewer #2

Gusain et al. characterized functional connectivity among bilateral frontal cortical areas using VSDI and electrical stimulation in mouse cortical slices. Notable findings were bilateral cortical activation elicited by direct white matter stimulation, and asymmetric functional connectivity between cg1 and cg2. These findings potentially provide important implications for the mechanism of activity propagation in epilepsy.

While the objective and the experimental approach of the study are reasonable, it was difficult to evaluate the credibility of the present findings because of the lack of description about some of the key analyses. This manuscript also lacks statistical assessment of the findings. Although some bar graphs have indications of statistical significance, I could not find any description of statistics (name of statistical test, p value, n etc.). I think this paper needs to be substantially improved in order to undergo proper peer review.

Major points

R: 1) Please add a description of statistics.

I could not find any description of statistics. Without statistics, it is not appropriate to claim that the results were “significant” (e.g. lines285 and 295). Some bar graphs contain asterisks that likely indicate statistical significance. Please describe the statistical testing (p values, statistics, n etc).

A: We apologize for the initial oversight and appreciate your patience. We have now updated the manuscript to provide a comprehensive statistical analysis. In the Materials and Methods section, we have included a description of the multiple comparison Tukey (HSD) test conducted using Igor Pro. Additionally, in the figure legends, we have incorporated detailed information, including p-values and sample sizes (n), to ensure clarity and reproducibility of the results. These amendments will better describe the statistical rigor of our study.

2) How did the authors define propagation ratio and activation?

In Figure 5D, authors use propagation ratio to quantify the difference between surface vs deep stimulation. I could not find a description of the method. Also, I could not find the definition of the activation. How did the authors define whether some pixels were activated or not activated? These points are essential for assessing the credibility of the main findings.

A: We appreciate your careful attention and questions regarding our methodologies. The “propagation ratio” is defined as the ratio of the response amplitude at the most dorsal side (upper dashed line in Fig. 5C) compared to that at the ventral side (the other dashed line in Fig. 5C) of the ACC. A ratio close to 1 indicates that the size of the response remained consistent to the end of the ACC.

3) How was the strength of electrical stimulation determined?

The results of functional connectivity should depend on the strength of stimulation. Ideally, the authors could test multiple stimulation strengths and characterize/interpret the functional connectivity as a function of stimulation strength. If this is not possible, the authors should add a discussion to validate their choice of stimulus strength.

A: We appreciate your question regarding determining stimulation strength. In our study, the electrical stimulation strength was set at {plus minus}40 V, delivered bipolarly for 500 μs each pulse. This intensity was carefully chosen based on previous experiences and considerations to obtain a slightly supramaximal response in the hippocampal slices, ensuring that we consistently activated the targeted pathways. The goal was to minimize variability that could arise due to differences in slice preparations and maintain consistency in our results. We hope this clarification addresses the reviewer’s concern, and we are open to further suggestions or discussion on this aspect.

Minor points

Line 50: “256 x 256 pixels” ?

A: Thank you for catching that detail. We appreciate the careful attention to the technical specifications in our manuscript.

Line 111-112: I think calling VSDI a “cutting edge” technique is a bit misleading. VSDI has been around for more than 20 years.

A: We appreciate your feedback and agree with you. The term “cutting-edge” has been removed to represent the maturity and establishment of the voltage-sensitive dye imaging (VSDI) technique more accurately in neuroscience research.

Lines140-146: I am not an expert of VSD imaging, but I found this VSD (Di-4 ANEPPS solution?) different from commonly used VSDs (e.g. RH1691 by Grinvald’s group). It would be helpful for readers if you cite previous publication(s) that characterize the Di-4 ANEPPS solution for VSDI.

A: Thank you for your insightful comment. We agree that it is essential to elucidate the rationale behind our choice of voltage-sensitive dye (VSD) for this study. RH1691 is indeed a commonly used VSD, particularly favored in in vivo experiments where dye wash-out is not a significant concern, but minimizing the overlap of fluorescent signal with intrinsic signals is crucial. RH1691’s fluorescent characteristics make it particularly suitable in such contexts.

In contrast, our study used in vitro experiments with slice preparations, where the stability of the dye within the tissue becomes a paramount consideration. Di-4-ANEPPS exhibits favorable characteristics in this regard, displaying a prolonged retention in the slices, lasting up to 12 h, which mitigates the issue of dye wash-out. This attribute of Di-4-ANEPPS is particularly advantageous for our experimental design, thus informing our decision to use it as the VSD in this study. We have added the necessary citations to our previous work in the Materials and Methods section of the manuscript.

R: Lines230-231 states that stimulation of white matter (S4,S5,S6) did not produce a clear response. But, I think S4 in Fig3B shows a clear response. Please explain.

A: Thank you for your meticulous observation and the opportunity to clarify this aspect of our study. We apologize for any confusion caused by our initial explanation. You are correct, and upon review, we recognize that our representation in Figure 3 may not have been explicit enough. Specifically, in the dataset illustrated, stimulation at points S5 and S6 in the white matter (particularly the corpus callosum) did not effectively propagate neural activity. Conversely, stimulation at point S4 did instigate discernible cortical activation, as visibly demonstrated in Figure 3A and further corroborated by the averaged data in Figure 4.

We acknowledge that while white matter stimulation (S4, S5, S6) can induce cortical activity propagation, the likelihood of induction appears relatively diminished, requiring a higher threshold compared to direct cortical stimulations. This is attributed to inherent variances in the activation thresholds between these distinct regions.

To improve clarity and precision in conveying this information, we have made necessary revisions to the manuscript. Again, we express our gratitude for your insightful feedback, which has been instrumental in enhancing the accuracy and comprehensibility of our presentation.

Figure 3B: Black numbers on blue background are very difficult to see. Please change the color.

A: Thank you for your suggestion. We have updated Figure 3B to improve visibility. The numbers are now displayed in a color that enhances contrast and readability against the blue background. We appreciate your attention to detail, ensuring that our visuals are as clear as possible.

Figure 4A shows substantial across animal variability. Please explain possible reasons.

A: We appreciate your attention to detail and the opportunity to address the variability observed in Figure 4A. We acknowledge the presence of substantial across-animal variability in our results, which may be attributed to multiple factors inherent in the delicate process of preparing brain slices.

Firstly, despite our meticulous efforts, there might be slight variations in the physiological conditions of the slices. Factors such as slight differences in temperature, oxygenation, and the overall health of the tissue can introduce variability in the response to stimulation.

Secondly, inherent complexities in the anatomical precision during slicing also play a role. Minor differences in the anatomical location from which slices are acquired, as well as slight deviations in the angle of slicing, can impact the uniformity of the responses observed across different animals.

We have strived to minimize these sources of variability by adhering to a consistent and careful slicing protocol and ensuring the best possible maintenance of slice physiology. The average map presented represents our best effort in consolidating the data while acknowledging these inherent variabilities. Thank you for allowing us to clarify this aspect of our study.

R:Figure 4 lacks a colorbar indicating the activity level.

A: Thank you for catching that omission. A color bar indicating the activity level has now been added to Figure 4, providing a clearer representation of the data. We appreciate your attention to detail, which enhances the clarity and comprehensibility of our figures.

Figure 7A lacks indication of statistical significance, but the main text states significant difference across slices. Which one is true?

A: Thank you for bringing this to our attention. There seems to have been an oversight in Figure 7A, and we apologize for the confusion. The text correctly states our findings; there is indeed a significant difference across slices. To rectify this, we have now updated Figure 7A to accurately reflect the statistical significance of the differences observed, as per the description in the text.

Figure 8D,E: Where is the “aberrant callosal projection”? Please indicate by arrows.

Also, is Figure8E a magnified view of the white rectangle in Figure 8D? They do not seem to match (Fig8E has square shape).

A: We appreciate your keen observation and apologize for any confusion caused by the presentation of our findings in Figure 8D and E. Your feedback has been instrumental in improving the clarity and accuracy of our illustration and corresponding text.

We have revised the figures and the manuscript to enhance clarity. In response to your observation, we have removed the term “aberrant” and refined our description to represent our findings more accurately. Furthermore, we apologize for any discrepancy in the alignment of the regions highlighted in Figure 8D and E. The necessary corrections have been made to ensure that Figure 8E accurately reflects a magnified view of the specified region within Figure 8D.
